# Metabolic Flux-Based Modularity using Shortest Retroactive distances

**DOI:** 10.1186/1752-0509-6-155

**Published:** 2012-12-27

**Authors:** Gautham Vivek Sridharan, Michael Yi, Soha Hassoun, Kyongbum Lee

**Affiliations:** 1 Department of Chemical and Biological Engineering, Tufts University, 4 Colby Street, Room 150, Medford MA 02155, USA; 2 Department of Computer Science, Tufts University, Medford, MA, USA

**Keywords:** Modularity, Metabolic networks, Metabolic flux, Edge-weighting, Adipocyte metabolism, Retroactivity

## Abstract

**Background:**

Graph-based modularity analysis has emerged as an important tool to study the functional organization of biological networks. However, few methods are available to study state-dependent changes in network modularity using biological activity data. We develop a weighting scheme, based on metabolic flux data, to adjust the interaction distances in a reaction-centric graph model of a metabolic network. The weighting scheme was combined with a hierarchical module assignment algorithm featuring the preservation of metabolic cycles to examine the effects of cellular differentiation and enzyme inhibitions on the functional organization of adipocyte metabolism.

**Results:**

Our analysis found that the differences between various metabolic states primarily involved the assignment of two specific reactions in fatty acid synthesis and glycerogenesis. Our analysis also identified cyclical interactions between reactions that are robust with respect to metabolic state, suggesting possible co-regulation. Comparisons based on cyclical interaction distances between reaction pairs suggest that the modular organization of adipocyte metabolism is stable with respect to the inhibition of an enzyme, whereas a major physiological change such as cellular differentiation leads to a more substantial reorganization.

**Conclusion:**

Taken together, our results support the notion that network modularity is influenced by both the connectivity of the network’s components as well as the relative engagements of the connections.

## Background

The topology of interactions in a biological network is often studied by modeling the network as a graph, which allows the use of established algorithms and metrics such as shortest path analysis [1] and betweenness centrality [2]. Graph theoretical models have yielded useful insights into not only the global topology of biological networks, but also local interactions that form distinct substructures, frequently referred to as modules [3,4]. Indeed, there is growing consensus that many types of biological networks possess modular character. Hierarchically arranged modules have been identified in metabolic networks, where larger, more heterogeneous subnetworks comprise smaller, more cohesive subnetworks [5,6]. Hierarchical modularity has also been observed for gene interaction networks [7] and protein interaction networks [8].

Despite the important insights obtained from topological analysis, almost all of the graph-based studies to date have examined a biological network under a single static condition [9]. For instance, Potapov and coworkers note that shortest path analysis, applied to a static network, may offer limited information because the length of an edge in the graph model may not correlate well with the overall efficiency of a particular biochemical transformation represented by the edge [10]. There is increasing evidence that biological network organization is dynamic and state dependent, which cannot be adequately studied from a static point of view. As a result, there has been growing interest in augmenting the topological information of biological networks for graph-based analysis with observed activity data. Recently, Tang and coworkers used gene expression data to construct time-course protein interaction networks, and found that functional modules detected in the time-course networks more closely matched known regulatory complexes than those detected in the static networks [11]. In another example, Greenblum and coworkers constructed a metagenomic network of the human gut microbiome using gene expression data, and showed that state-specific networks representing lean or obese individuals exhibited different topological properties, including modularity [12]. Similarly, Taylor and coworkers found that dynamic changes in the organization of the protein-protein interaction network, rather than expression levels of individual proteins, correlated strongly with breast cancer prognosis [13]. Interestingly, mutations in hub proteins connecting different modules were found to be more frequently associated with cancer phenotypes than mutations in hub proteins that are highly connected with other proteins in the same modules, suggesting that alterations in global modularity may occur in cancer.

In the case of a metabolic reaction network, gene or even protein expression data may not best capture the interactions between the network’s components, as mRNA levels or enzyme concentrations do not necessarily correlate with reaction rate or metabolite turnover. A more comprehensive snapshot of the physiological state may be provided by a metabolic network’s reaction flux distribution, which directly reflects the relative engagements of enzymes, integrating the various layers of regulatory processes active in the cell. Intuitively, the flux of a reaction can be used to weight the interaction mediated by the reaction. For example, Yoon and coworkers applied flux-based weights to adjust the edge distances in a graph model of murine adipocyte metabolism, and thereby reflect metabolic state-dependent variations in the interactions between metabolite pools [14,15]. While intuitive, this weighting scheme assumes that the metabolic network is modeled as a metabolite centric graph, where the edges represent reactions. For the purpose of studying the interactions between enzymes, it is often useful to model the metabolic network as a reaction centric graph, where the nodes represent enzymes and edges represent interactions between the enzymes mediated by metabolite substrates and effectors [16]. The benefit of a reaction-centric graph, particularly in the context of modularity analysis, is that a metabolite is not constrained to a module. Instead, a metabolite is more appropriately modeled as a shared resource, and reactions define the functional identity of a module. To our knowledge, a scheme to weight the edges of a reaction-centric graph has not yet been described in the literature. The purpose of this study was therefore to develop a generally applicable method for incorporating activity data such as metabolic flux into modularity analysis using graph models where the nodes, rather than the edges, represent the network’s functional components.

Recently, we defined a new metric, termed Shortest Retroactive Distance (ShReD), to capture feedback and other cyclical interactions in a metabolic network [17]. Based on the earlier work of Saez-Rodriguez and coworkers on retroactivity [18], ShReD was used to solve for modular partitions that would minimize cyclical interactions between modules while maximizing such interactions within a module. While the earlier work on retroactivity focused on nearest neighbor interactions, for example mediated by the product of a reversible reaction, the ShReD-based analysis also considered interactions between distant parts of a network. In the present study, we further expand the use of ShReD as a modularity analysis metric by developing a weighting scheme to reflect phenotypic state-dependent variations in reaction-to-reaction interactions. We focus on flux data due to the integral nature of the information content in such data, reflecting the functional outcomes of transcriptional, translational, and post-translational mechanisms of enzyme activity regulation. Flux data can be obtained using a number of different methods, including isotopic (typically ^13^C) labeling, metabolic flux analysis (MFA), and flux balance analysis (FBA), Generally, mathematical model-based analysis of isotopic enrichment of multiple metabolite pools offers the greatest resolution. Flux balance analysis is a constrained optimization based approach typically used to estimate fluxes in conjunction with a metabolic objective function. The problem is usually severely underdetermined in FBA. In the present study, we used a constrained optimization based approach to estimate metabolic fluxes, but without assuming a metabolic objective. Rather, we minimized the sum of squared differences between the measured and estimated exchange fluxes, as the problems were well constrained. Applied to a model of adipocyte metabolism, ShReD-based modules obtained using flux weights more consistently reflected recognizable functions of established pathways compared to the modules obtained without the weights. Comparisons of modules obtained using several different flux sets representing distinct metabolic states identified robust reaction pairs that repeatedly partitioned into the same module across many levels of modular hierarchy, suggesting possible co-regulation.

## Results

### Effects of weighting edges on ShReD distribution

**Figure 1:**
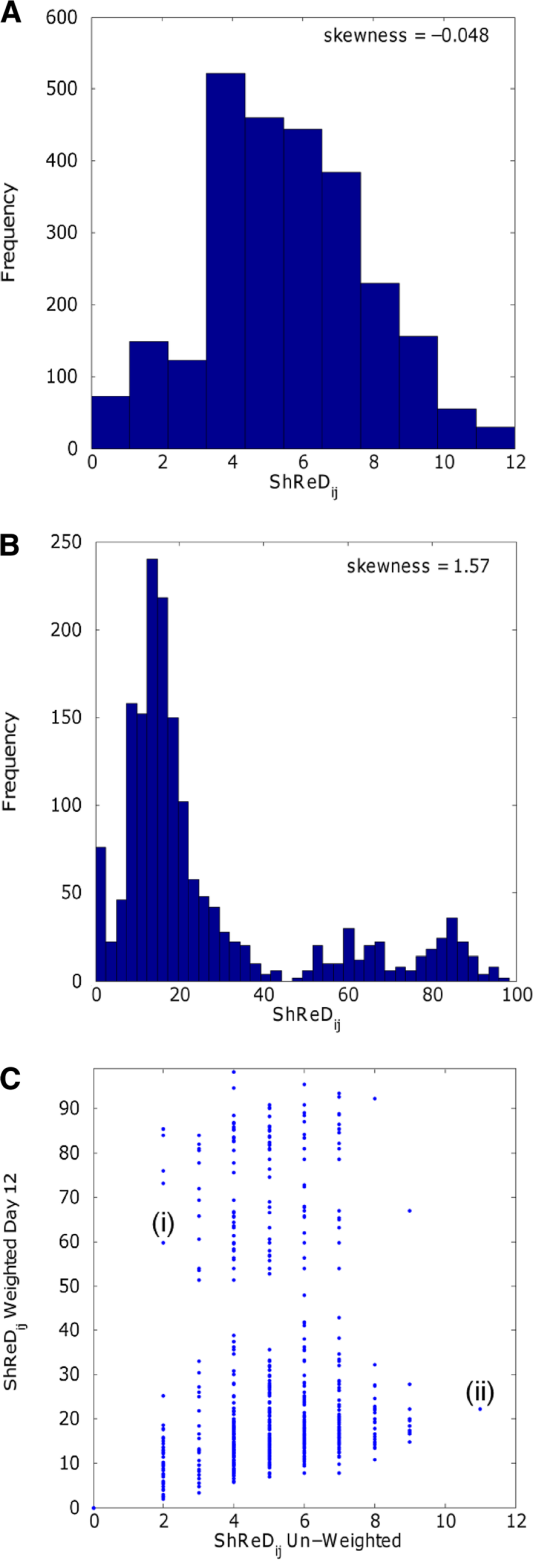
**(A) Distribution of ShReD values for the un-weighted adipocyte network.** The histogram only shows ShReDs with value < 100. (**B**) Distribution of ShReD values for the Day 12 flux-weighted model. The histogram only shows ShReDs with value <100. (**C**) Scatter plot of Day 12 weighted ShReD and un-weighted ShReD for all reaction pairs [i, j]. The plot only shows reaction pairs with ShReD value < 100. Points (i) and (ii) represent reaction pairs [R28, R63] and [R42, R50], respectively.

Weighting the edges that define the interactions between the reaction nodes substantially impacted the overall distribution of ShReDs. In the un-weighted case, when all edges have the same unit distance, the ShReDs are close to normally distributed, with a skewness of −0.048 (Figure 1A). In the weighted cases, the distributions clearly skew to the right. For example, the skewness of the Day 12 model is 1.57 (Figure 1B). This difference in ShReD distribution between the weighted and un-weighted cases motivated an adjustment from our earlier modularity metric. In our previous work, the entries for the modularity matrix were computed as the difference between the expected and actual ShReD of two reaction nodes, where the expected ShReD was calculated as the arithmetic average of all ShReDs involving either one of the two reaction nodes [17]. This calculation assumed that the overall ShReD distribution is close to normal, and that the arithmetic average reasonably represents the expected ShReD between two nodes. With the incorporation of reaction flux-based weights, many reaction-to-reaction interactions were orders of magnitude weaker (and the corresponding graph distances were longer) than the average interaction, for example, due to the involvement of amino acid reactions whose fluxes were negligible compared to those of glucose and lipid metabolism. Therefore, in this study we introduce a modified modularity matrix **V,** which ranks the ShReD between two reaction nodes relative to the distribution of all ShReDs involving either one of the two reaction nodes.

There is a positive correlation (R^2^=0.35, p<0.01) between the un-weighted ShReD and the corresponding weighted ShReD. The correlation analysis was performed on reaction pairs with ShReD < 100, since the maximal ShReD value was capped at 100 (see Methods). The positive correlation suggests that the topology of the metabolic network as defined by the stoichiometry has some influence on the closeness of cyclical interactions between enzymes as defined by the fluxes of the reactions connecting the enzymes (Figure 1C). However, the correlation is not very strong, as there are many instances where a relatively short un-weighted ShReD corresponds to a relatively long weighted ShReD (Figure 1C, i), and a long un-weighted ShReD corresponds to a short weighted ShReD (Figure 1C, ii).

### Effects of edge-weighting on ShReD-based network partition

**Figure 2:**
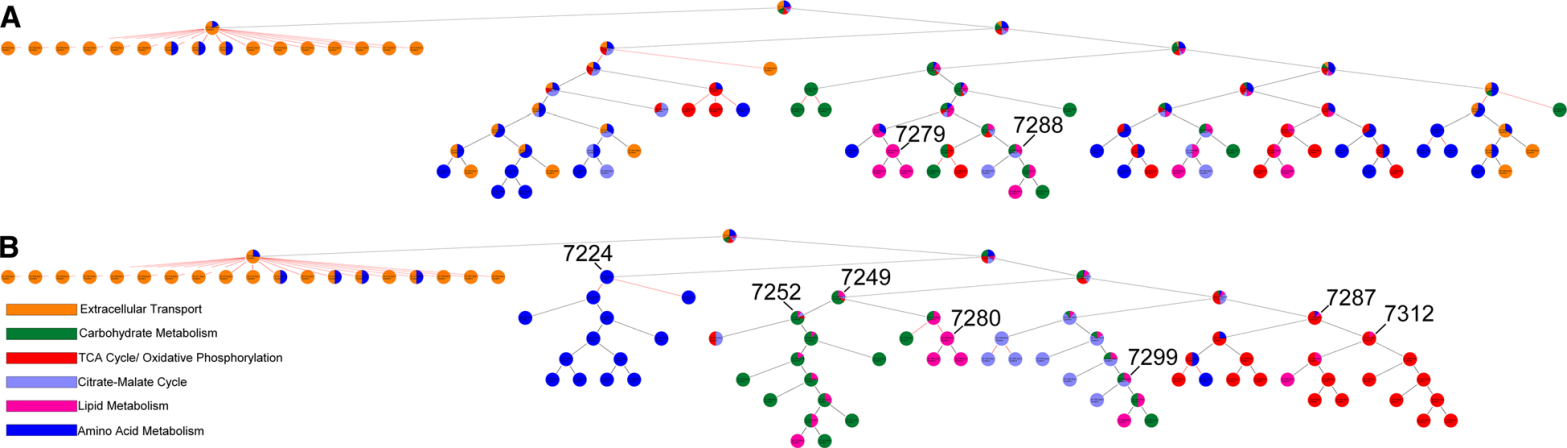
**(A) ShReD-based partition for the un-weighted model. (B)** ShReD-based partition for the Day 12 flux-weighted model. The partition trees represent each module as a pie chart, where the size of each slice is proportional to the fraction of reactions in the module that belong to the corresponding, pre-assigned canonical group. The group assignments are indicated by color as shown in the legend. Modules referred to in the text are labeled with identification numbers. Reactions associated with each module are listed in Additional file 2.

The ShReD-based hierarchical partition of modules for the un-weighted adipocyte model is compared to the partition for the flux-weighted Day 12 model (Figure 2). There are striking similarities between the two partitions. In both cases, the transport reactions and a few amino acid metabolism reactions peel off from the original network after the first partition. Additionally, several key interactions between reactions are conserved. The partitions point to close interactions between carbohydrate metabolism, lipid metabolism, and citrate-malate cycle for both the un-weighted case (Figure 2A: Module #7288 for module assignment details, see Additional File 2) and the flux-weighted case (Figure 2B: Module #7299). Similarly, there is tight coupling between triglyceride synthesis and degradation that persists through multiple partition levels for both cases (Figure 2A: Module # 7279, Figure 2B: module #7280).

**Figure 3:**
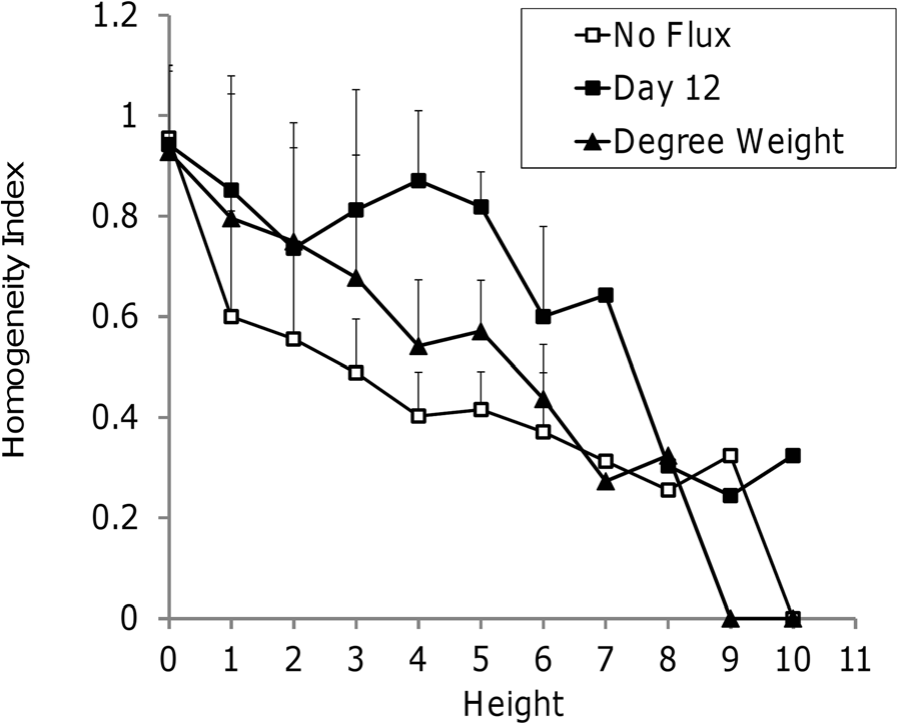
Average homogeneity index of the modules as a function of partition height (see Methods for definition of homogeneity index).

However, there are also several qualitative differences between the two partitions. The un-weighted partition broadly distributes TCA cycle and amino acid reactions across various branches in the hierarchical partition tree (Figure 2A). However, in the flux-weighted case, the *a priori* assigned textbook associations largely remain intact (Figure 2B: Module #7224, Module #7287). Quantitatively, the flux-weighted partition has a greater average homogeneity index between heights 1–7 in the hierarchy (Figure 3), where height zero corresponds to terminal nodes. At height zero, the average homogeneity is similarly high for both the weighted and un-weighted cases due to the large number of single reaction modules.

### Comparing dynamic vs. Static weighting schemes

In the absence of flux data, topological data other than cyclical connectivity could be used to guide modularity analysis. Metabolite degrees [19] were investigated as an example of connectivity-based weights reflective of network topology from a static perspective. Briefly, the edge distance from a reaction node R_i_ to reaction node R_j_ was determined as the number of reactions in the network that consume the intermediary metabolite connecting R_i_ and R_j_. The rationale was that the influence of R_i_ on R_j_ would be strongest if R_j_ is the only reaction consuming the intermediary metabolite produced by R_i_. The influence would be weaker if the intermediary metabolite was consumed not only by R_j_, but also by many other reactions in the network. Applying this weighting scheme to the adipocyte model (Figure 3), we find that ShReD-based partitioning of the metabolite degree-weighted network results in average homogeneity index values that lie between the un-weighted network and the flux-weighted network. This result suggests that the metabolite degree-weighted network is an improvement over the un-weighted network, but is less effective than the flux-weighted network at capturing the relative engagements between the reactions.

### Robust interaction pairs

**Figure 4:**
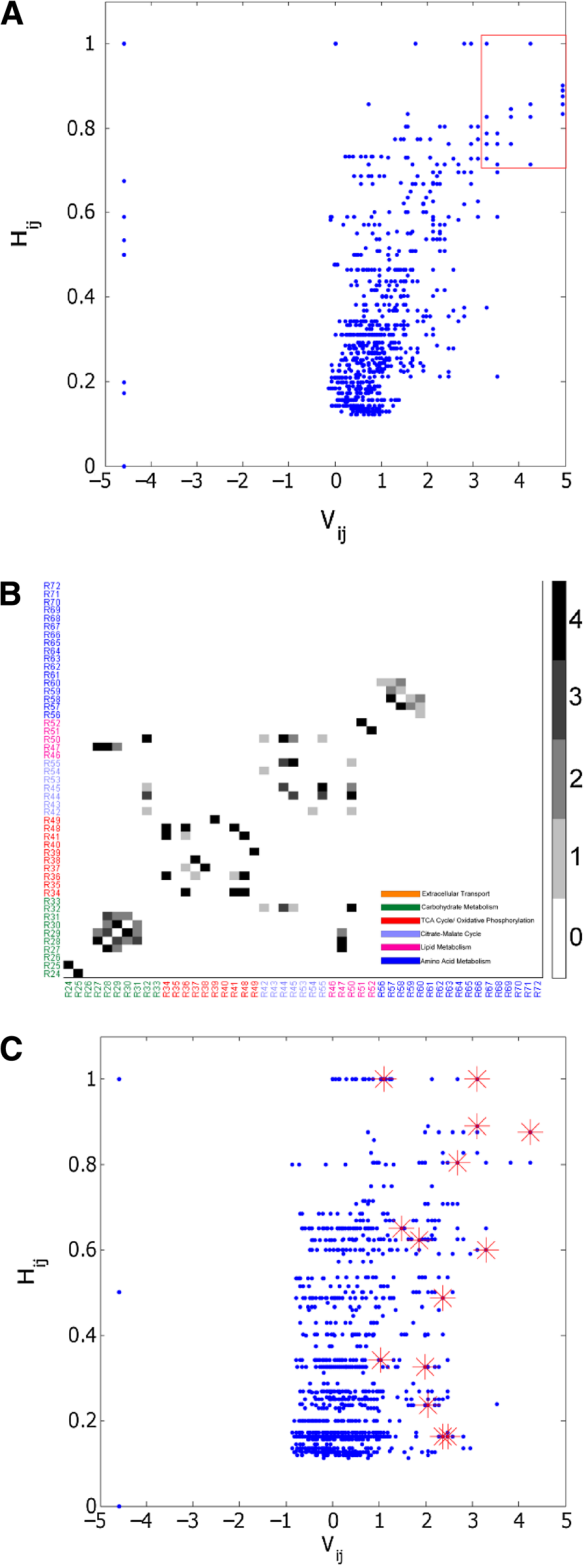
**(A) Scatter plot of partition score H**_**ij**_**and modularity score V**_**ij**_**for all reaction pairs [i, j] in the Day 12 flux-weighted model.** A red box is drawn around reaction pairs with H_ij_ > 0.7 and V_ij_ > 3.0. (**B**) Heat map showing the number of models (Day 4, Day 12, Day 12 with LDH inhibition, Day 12 with PCX inhibition) for which a reaction pair [i, j] has a high partition score and a high modularity score (H_ij_ > 0.7 and V_ij_ > 3.0). A reaction pair was designated as robust if H_ij_ > 0.7 and V_ij_ > 3.0 in all four models. The robust reaction pairs are shown as black squares in the heat map. (**C**) Scatter plot of partition score H_ij_ and modularity score V_ij_ for all reaction pairs [i, j] in the un-weighted model. The red asterisks denote the 17 robust reaction pairs identified in panel (**B**).

We next investigated whether the modularity score V_ij_ of two reaction nodes in the initial un-partitioned network could predict the degree to which the two reaction nodes remain together in the hierarchical partitioning. The degree to which two reaction nodes remain together was assessed by the partition score H_ij_, which scales the number of modules shared by both reaction nodes with respect to the total depth of the partitions for each reaction node (see Methods for definition of depth). A scatter plot of the partition score and the modularity score for the Day 12 flux-weighted model shows a significant positive correlation (R^2^ = 0.45, p<0.01) for reaction pairs with a positive modularity score (Figure 4A). Of particular interest are the reaction pairs that fall in the upper right hand corner, chosen here to be reaction pairs with V_ij_ > 3.0 and H_ij_ > 0.7. Reaction pairs satisfying this criterion were selected from all four flux-weighted adipocyte models (Day 4, Day 12, and Day 12 with PCX or LDH inhibition). Forty reaction pairs, or roughly 1.5% of the possible 2556 reaction pairs, satisfied the criterion for at least one of the four models. A heat map displaying the number of models (of the four adipocyte models) for which a given reaction pair meets the criterion shows that 17 of the 40 reaction pairs robustly partition together across the different metabolic states (Figure 4B). One such reaction pair is [R32, R50] (for reaction definitions, see Additional file 1: Table S1), which corresponds to NADPH production from the pentose phosphate shunt and NADPH consumption for palmitate synthesis, respectively. To determine whether these robust reaction pairs could be identified solely based on stoichiometry in the absence of flux information, each of the 17 reaction pairs were mapped onto a corresponding plot of modularity and partition scores for the un-weighted adipocyte model (Figure 4C). Overall, the correlation between the partition and modularity scores, albeit still significant, was weaker for the model without flux weights (R^2^=0.11, p<0.01). Only 5 of the 17 robust reaction pairs identified in Figure 4B have partitions scores > 0.7, and only 3 reaction pairs also have modularity scores > 3.0 in the un-weighted H-V plot. The three reaction pairs are [R34, R36] corresponding to pyruvate dehydrogenase/citrate synthase and isocitrate dehydrogenase in the TCA cycle, [R51, R52], corresponding to triglyceride synthesis and degradation and [R57, R58], corresponding to glutamate synthesis and degradation. The remaining robust reaction pairs identified in the four flux-based partitions are not found in the un-weighted network partition. For example, the robust pair [R29, R30], which corresponds to reactions in glycolysis, has relatively low partition and modularity scores in the un-weighted case.

### Impact of metabolic state on modularity

**Figure 5:**
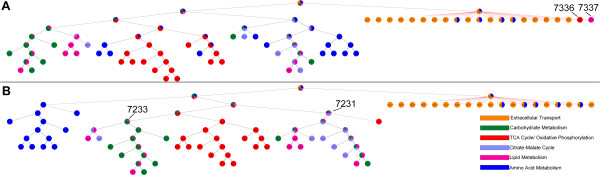
**(A) ShReD-based partition for the Day 4 flux-weighted model.** (**B**) ShReD-based partition for the Day 12 model with LDH inhibition. The partition trees represent each module as a pie chart, where the size of each slice is proportional to the fraction of reactions in the module that belong to the corresponding, pre-assigned canonical group. The group assignments are indicated by color as shown in the legend. Modules referred to in the text are labeled with identification numbers. Reactions associated with each module are listed in Additional file 2.

The partitions of the four flux-weighted models show clear differences in hierarchical modularity. The differences are mainly due to the placement of the reactions catalyzed by phosphoenolpyruvate carboxykinase (PEPCK) and pyruvate carboxylase (PCX). For the Day 4 model, the first partition of the initial network isolates both enzymes from the main network along with transport reactions that do not have any cyclical interactions (Figure 5A: Modules #7337, 7336 respectively). For the Day 12 model, PEPCK is tightly coupled to the TCA cycle reactions (Figure 2B, Module #7312), and PCX is coupled to carbohydrate metabolism (Figure 2B, Module #7252). For the LDH-inhibition model, PEPCK is coupled to carbohydrate metabolism reactions (Figure 5B, Module #7233) while PCX is coupled to triglyceride metabolism and reactions that produce NADPH (Figure 5B, Module #7231).

**Figure 6:**
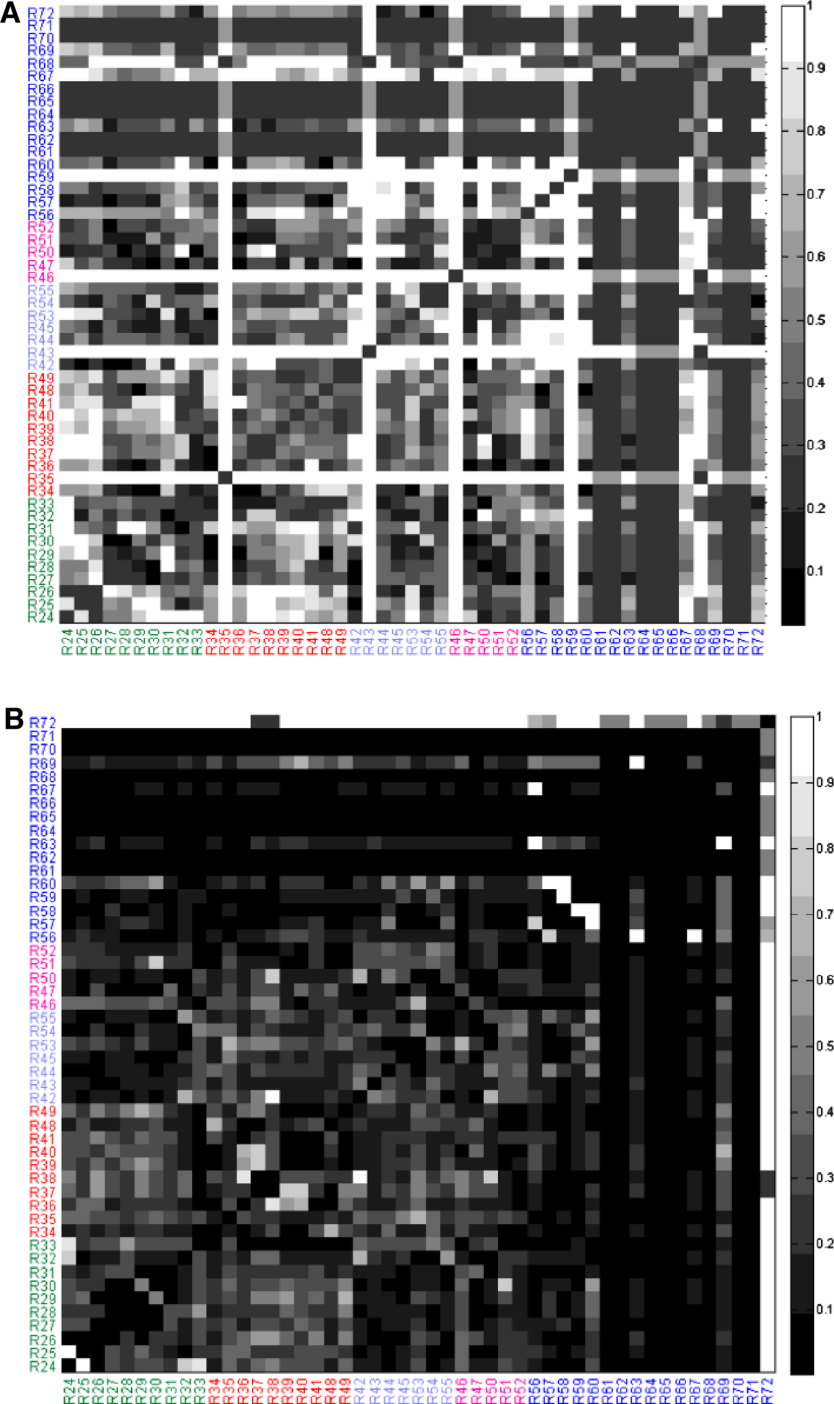
**(A) Heat map of the H-V Euclidean distance between the Day 4 model and Day 12 model (see Methods for definition of distance).** (**B**) Heat map of the H-V Euclidean distance between the Day 12 model and Day 12 model with LDH inhibition. The colors for the reaction numbers correspond to the same group assignments as in Figures 2 and 5.

To quantitatively assess and visualize the overall impact of metabolic state on the hierarchical partitioning of modules, we computed the Euclidean distance a reaction pair moves in the normalized H-V space of a flux-weighted model relative to another flux-weighted model (See Methods). A large distance implies that the reaction pair’s partition and modularity scores (the coordinates of the H-V space) are substantially influenced by the change in metabolic state, whereas a short distance suggests that the change in metabolic state has little impact on the reaction pair’s placement in the module hierarchy. These distances are shown as heat maps for the changes in flux distribution between Day 4 and Day 12 (Figure 6A) and Day 12 and Day 12 with LDH inhibition (Figure 6B). For both models, the heat maps feature several dark regions (reflecting very little movement). These regions largely correspond to reaction pairs involving amino acid metabolism, but also include reaction pairs involved in carbohydrate metabolism (e.g. [R27, R30]), and the TCA cycle (e.g. [R39, R41]). Overall, it is clear that the change in flux distribution between Day 4 and Day 12 has a more pronounced effect on modularity compared to the change in flux distribution between Day 12 and Day 12 with LDH inhibition. A similar observation was made for the change in flux distribution between Day 12 and Day 12 with PCX inhibition, which also had a smaller impact on modularity compared to the change in flux distribution between Day 4 and Day 12 (Additional file 1: Figure S1).

## Discussion

In this paper, we present a novel methodology for investigating the impact of different metabolic states on the functional organization of metabolic networks. The methodology utilizes metabolic flux data as weights for a graph-based partitioning method that conserves cyclical interactions. Previously, we assessed the cyclical interactions based on ShReDs calculated by assuming static interactions, and thus a uniform graph distance, between each connected reaction pair. In the present study, we allow the interactions, and thus the graph distances, to vary with the metabolic state.

Unlike the un-weighted case, the weighted ShReD distribution displays significant skewness (Figure 1B), indicating that the arithmetic mean is not representative of the average or expected ShReD. A likely reason for the skewness is that some reactions, particularly those involved in amino acid metabolism, carry negligible flux compared to other parts of central carbon metabolism such as glycolysis and the TCA cycle. As the edge weights of a ShReD are inversely proportional to the fluxes of the reactions comprising the ShReD, a ShReD that includes one or more reactions carrying negligible flux can be very large, and thus skew the arithmetic mean of the distribution. For this reason, it is possible for a reaction pair to have a relatively small ShReD in an un-weighted network, but a relatively large ShReD in a corresponding weighted network. For example, the ShReD for the reaction pair [R28, R63] in the unweighted network is two (2) (Figure 1C, i), since R28 (3-phosphoglycerate synthesis in glycolysis) and R63 (proline synthesis) interact cyclically via the production and consumption of NADH and NAD^+^. However, in the weighted network, the directional interaction from R28 to R63 is very weak, since only a very small fraction of the NADH produced by glycolysis is used for proline synthesis. The corresponding edge distance is 1251, which is approximately 50-times the average of non-infinite ShReDs in the network (25). As a result, the weighted ShReD between these two reactions traverses an alternate sequence of reaction nodes, comprising 10 reactions spanning parts of glycolysis and the TCA cycle, 2-oxoglutarate synthesis, and glutamate synthesis (Additional file 1: Figure S2). The ShReD value of this cycle is ~60. This ShReD value is still relatively large compared to other weighted ShReD values in the distribution, implying a relatively weak cyclical interaction. Conversely, a relatively long ShReD in the unweighted network can yield a relatively short ShReD in the weighted network. For example, the unweighted ShReD for the reaction pair [R40, R52], corresponding to mitochondrial malate synthesis and triglyceride degradation respectively, is the largest non-infinite ShReD at 11 (Figure 1C, ii). However, every edge in this cycle carries a relatively large flux, resulting in a weighted ShReD value of 22, which is close to the average ShReD of the weighted network.

A comparison of the hierarchical partition trees for the unweighted and weighted (Day 12) models shows that the weighted model yields greater functional homogeneity of modules based on the canonical pathway assignments of the constituent reactions (Figures 2 and 3). This suggests that the network topology alone, as defined by the network’s reaction stoichiometry, is insufficient to capture the functional associations between reactions that are reflected in the textbook pathway assignments. In our previous work, we augmented the stoichiometric information by including known regulatory interactions between reactions. Edges denoting regulatory interactions were drawn from one reaction node to another if the product metabolite of the first reaction allosterically regulated the second reaction. The presence of these regulatory edges had a significant impact on the modularity of the network. However, for many cell types, information regarding regulatory mechanism is incomplete or difficult to obtain, requiring extensive manual searches of the literature. Therefore, an un-weighted network will almost certainly contain only partial information regarding functional interactions between reactions. One way to upgrade the information content is to incorporate metabolic flux data, which provides a snapshot of cellular metabolic state, and reflects the integral of various regulatory processes active in the cell. In this study, we found that incorporating flux data as weights for directed interactions between reactions resulted in homogeneous modules that are more in line with textbook knowledge on biochemical pathway organization.

However, we found that some module inhomogeneity persists deep into the hierarchy even for the weighted models. A majority of these inhomogeneous modules include one or more robust reaction pairs that consistently partition together across the different metabolic states examined in this study. One such module, found at depth 7 of the Day 12 model partition (Figure 2B, Module #7299), points to a tight coupling between carbohydrate metabolism, citrate malate cycle, and lipid metabolism, mediated through the production and consumption of NADPH. This module includes the reaction pair [R32, R50], corresponding to NADPH production via the pentose phosphate shunt and palmitate synthesis respectively, which was one of the 17 robust reaction pairs with both a high modularity score and a high partition score for all four flux-weighted partitions. We have previously observed that the interactions mediated by cofactors, which are ubiquitously present throughout metabolism, can couple reactions spanning seemingly distant pathways [17]. Prior studies have often removed cofactors or ‘currency metabolites’ prior to network modularization due to the difficulty of assigning them to distinct functional modules. While ShReD-based partitioning can also be performed after the removal of cofactors, our prior work suggests that cofactors are essential in mediating metabolic cycles and allosteric feedback loops [17], and should thus be retained if the goal is to identify modules based on cyclical interactions.

One possible biochemical basis underlying the robust reaction pairs is co-regulation. For example, reaction R50, catalyzed by 3-oxoacyl-(acyl-carrier-protein) reductase, requires NADPH as a cofactor for activity [20], while both enzymes catalyzing the lumped reaction R32, glucose 6-phosphate dehydrogenase and 6-phosphogluconate dehydrogenase, are allosterically regulated by NADPH [21,22]. Similarly, reaction R44, catalyzed by malic enzyme, is product-inhibited by NADPH [23], which is a required cofactor for reaction R50. Another co-regulated robust reaction pair is [R34, R48], corresponding to the first steps in the TCA cycle (pyruvate dehydrogenase and citrate synthase) and oxidative phosphorylation, respectively. Oxaloacetate is a limiting substrate for citrate synthase, and also a competitive inhibitor of oxidative phosphorylation [24]. Reactions R34 (pyruvate dehydrogenase/citrate synthase), R36 (isocitrate dehydrogenase) and R41 (malate dehydrogenase) are steps in the TCA cycle regulated by ATP, which could explain the robustness of interactions between reaction pairs [R34, R36] and [R34, R41] [23,25,26].

While the partitions of the four flux-weighted models share similar modules as exemplified by the robust reaction pairs, they also exhibit notable differences. For the Day 4 partition, reactions catalyzed by PCX and PEPCK both split off immediately from the parent network at depth one of the hierarchy. This split is due to the very low flux carried by these reactions at Day 4, which excludes them from significant cyclical interactions with any of the other reaction nodes. Day 4 represents an early stage of differentiation when an immature adipocyte phenotype is expected. While lipogenic genes are activated, the fluxes of lipid synthesis and triglyceride accumulation remain low at this stage relative to other parts of central carbon metabolism. Our results suggest that PCX and PEPCK, which catalyze upstream steps in glycerogenesis and fatty acid synthesis from glucose, are not yet integral to any major functional modules in the immature adipocyte. However, at Day 12 (Figure 2B), PEPCK is tightly coupled to the TCA cycle reactions, mediated through the consumption and production of ATP, and PCX is coupled to carbohydrate metabolism and triglyceride metabolism (Figure 2B Module #7252). Indeed, there is a striking difference between Day 4 and Day 12 partitions based on the relative distances between the corresponding pairs of reactions in the H-V space. In comparison, there is a more subtle difference between the partitions of Day 12 and Day 12 with LDH inhibition. These observations suggest that the inhibition of one enzyme is not enough to drastically alter modularity. In contrast, the transition from an immature phenotype on Day 4 to a mature phenotype on Day 12 represents a concerted set of changes across cellular metabolism, which is reflected in the broadly altered modularity.

## Conclusions

Taken together, our results support the notion that network modularity is influenced by both the connectivity of the network’s components as well as the relative engagements of the connections. The major contribution of the present study is a generally applicable methodology to incorporate activity data into a systematic partitioning framework featuring the conservation of cyclical, or retroactive, interactions. We found two key benefits of incorporating metabolic flux data. First, comparisons across different metabolic states can identify conserved modules comprising robustly interacting reactions that may be co-regulated by a common allosteric effector. Second, embedded in the flux data is information on the various layers regulatory processes active in the cell, which can be used to augment connectivity relationships defined by stoichiometry. In the context of modularity analysis, the implication is that lack of detailed knowledge on regulatory mechanisms can be at least partially addressed using experimentally observable data. On the other hand, the reliance on experimental data is also a limitation in the scalability of our methodology. As modularity analysis is an approach to study complex networks, it is ideally applied to large-scale systems rich with complexity. Unfortunately, resolving the flux distribution of a large-scale metabolic network, for example using ^13^C isotope labeling, remains experimentally demanding and technically challenging. One way to address this limitation in scalability could be to utilize solutions from constraint-based methods such as Flux Balance Analysis that require relatively few measurements. Rather than rely on flux data reflecting an observed metabolic state, flux data could be used that reflect an optimized state or a range of attainable states. An added benefit of using such model-derived flux data could be to enable efficient exploration of different module configurations accessible to a metabolic network.

In summary, we have extended a previously developed methodology for modularity analysis by considering non-uniform interactions between retroactively connected reactions. Whether the modules defined by cyclical interactions between their constituent reactions indeed contribute to some recognizable system property warrants further study. For example, a future study could examine whether modules comprising metabolic cycles serve to limit the propagation of perturbations through the network, and thereby add to the stability of the system.

## Methods

### Adipocyte model and fluxes

A stoichiometric network model of adipocyte central carbon metabolism was formulated by slightly modifying a previously published model [15]. The modifications were as follows. Reactions were removed for ketone body metabolism, because these reactions carried negligible flux. Reactions were added for glyceroneogenesis to allow the synthesis of glycerone-phosphate from phosophoenolpyruvate. The number of reactions and metabolites in the modified model were 72 and 79, respectively, with 48 independent steady state balances and 22 measured exchange rates. The system was underdetermined by a degree of two. Metabolic flux distributions were calculated for four different phenotypic states: immature adipocyte (day 4 post-induction), mature adipocyte (day 12 post-induction), mature adipocyte treated with an inhibitor for lactate dehydrogenase (LDH), and mature adipocyte treated with an inhibitor for pyruvate carboxylase (PCX). Rates of metabolite uptake and output (exchange rates) describing these phenotypic states were taken from our previous work [15,27]. Fluxes were calculated by minimizing the sum of squared differences between measured and calculated metabolite exchange rates subject to stoichiometric balance constraints. The reaction definitions of the adipocyte model and flux distributions corresponding to the four phenotypic states are listed in Tables S1 and S2 (Additional file 1).

### Flux-based ShReD

**Figure 7:**
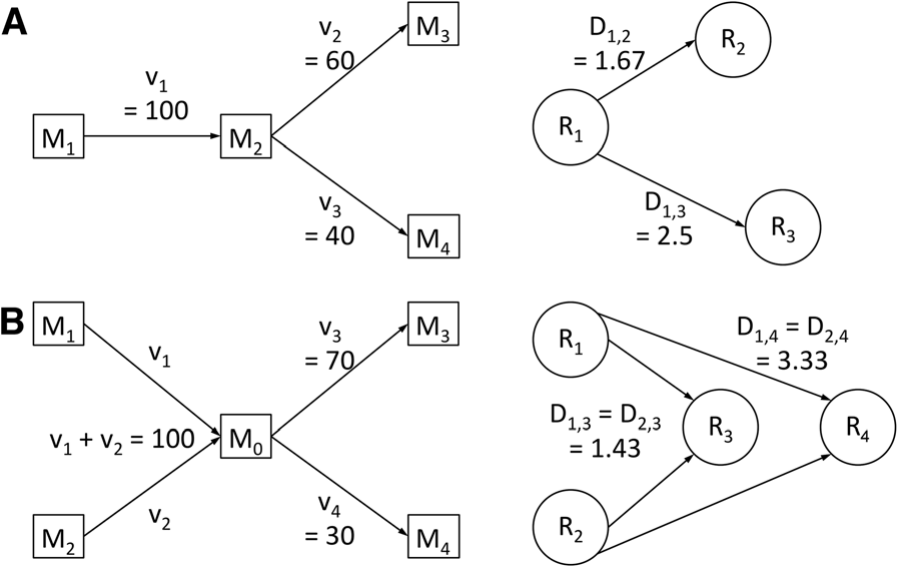
Examples of metabolic flux-based edge weighting in the case of (A) one reaction producing the intermediary metabolite and (B) multiple reactions producing the intermediary.

Previously, we defined a metric, termed ShReD, to characterize the connectivity between two biochemical network components that interact retroactively [17]. Connectivity relationships between reactions in a metabolic network as defined by stoichiometry can be modeled using a directed graph with vertices representing reactions and edges indicating a directional interaction between connected reactions. Edges are drawn between two reactions if the product of one reaction is a reactant of the other reaction. Based on this graph model, the ShReD of reaction nodes i and j is computed as the sum of the shortest path distance from node i to j and the shortest return path from node j to i. In computing the shortest path distance and shortest return path distance, each edge can be assigned the same unit distance to consider a nominal state where the interactions between reactions are solely determined by network topology. Alternatively, each edge can be assigned a different weight that reflects the engagement of the biochemical interaction represented by the edge. For a pair of metabolic reactions, a quantitative measure of this interaction can be obtained from the flux of the intermediary metabolite. Consider the example in Figure 7A. Reaction R_1_ produces 100 mol/min of metabolite M_2_, of which 60 mol/min is directed towards R_2_ and the remainder towards R_3_. Assuming that the pool of M_2_ is homogeneous, we attribute a stronger influence of R_1_ on R_2_ relative to R_3_. Intuitively, a stronger influence is modeled as a smaller edge weight (shorter path distance), whereas a weaker influence is modeled as a larger edge weight (longer path distance). Formally, we define the edge distance between a connected pair of reaction nodes as the *inverse of the fraction of the intermediate metabolite production flux that is directed towards the destination reaction node*. In the example of Figure 7A, the dimensionless edge distance D_1,2_ between R_1_ and R_2_ is given by:

(1) $$D_{1,2}=1/\left(60,/,100\right)=1.67$$

The edge distance between R_1_ and R_3_ is given by:

(2) $$D_{1,3}=1/\left(40,/,100\right)=2.50$$

The above definition can be extended in a straightforward manner to cases when more than one reaction produces the metabolite that mediates the interaction between two reactions (Figure 7B). These cases often involve energy cofactors such as ATP, NADH, or NADPH, which are produced and consumed by many reactions. In the example of Figure 7B, the total steady state flux of M_0_ is 100 mol/min, of which 70 and 30 mol/min is directed towards R_3_ and R_4_, respectively. Based on the assumption that the metabolite pool is homogeneous and the contributions of the upstream reactions to this pool are indistinguishable, the directed interaction from R_1_ to R_3_ is the same as the interaction from R_2_ to R_3_. The weighting would be the same if *v*_*1*_ and *v*_*2*_ are each 50, or if *v*_*1*_ is several orders of magnitude smaller than *v*_*2*_. Even if *v*_*1*_ = 0.01, it has to be assumed that 70% of that small flux is directed towards R_3_, because the source of the intermediary metabolite flux cannot be distinguished by the downstream enzymes. Similarly, the interaction from R_1_ and R_4_ is the same as the interaction from R_2_ to R_4_. Generalizing for a pair of reactions R_i_ and R_j_ connected through an intermediary metabolite M produced by an arbitrary number reactions N, the edge distance from node R_i_ to R_j_ is given by:

(3) $$D_{\mathrm{ij}}=\left(\sum_{k=N}v_{k}\right)/v_{j}$$

**Figure 8:**
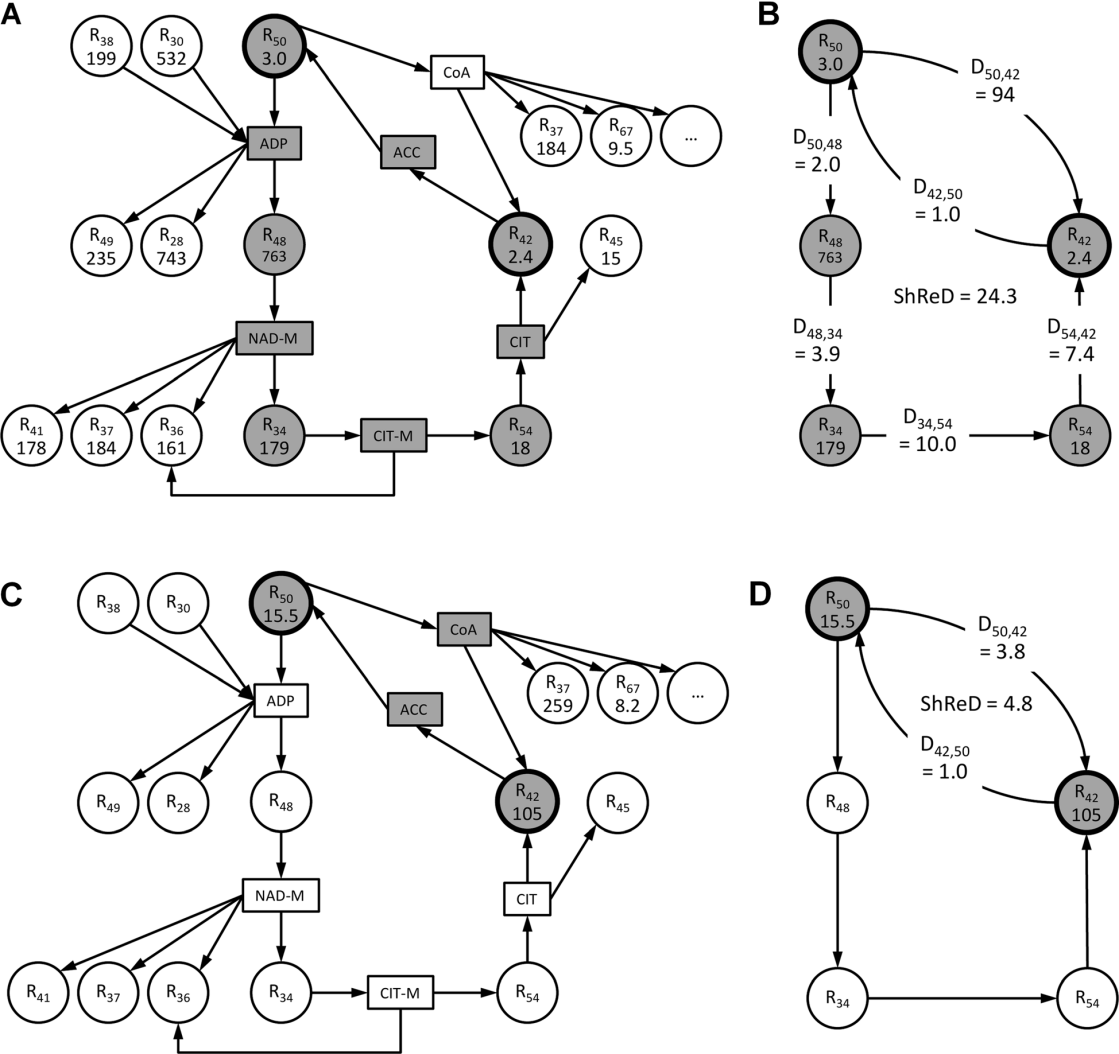
**Example illustrating the calculation of a ShReD based on flux weights.** Circles and boxes show reactions and metabolites, respectively. The top and bottom numbers in the circles refer to the reaction number and corresponding flux, respectively. The ShReDs are highlighted with grey circles and boxes. (**A**) Day 4 model flux distribution around reaction nodes comprising the ShReD for the reaction pair [R42, R50]. The reactions comprising the ShReD are as follows. R_50_: palmitate synthesis, R_48_: oxidative phosphorylation, R_34_: pyruvate dehydrogenase and citrate synthase, R_54_: mitochondrial transport of 2-oxoglutarate and malate, R_42_: citrate lyase. (**B**) Reaction-to-reaction distances calculated using the fluxes shown in panel (**A**) and equation 3. Note that the path from R_50_ to R_42_ in ShReD_42,50_ proceeds through R_48_, R_34_, and R_54_ due to the long weighted distance from R_50_ to R_42_, which in turn is due to the relatively small contribution of R_50_ to the CoA flux. (**C**) Day 12 model flux distribution around reaction nodes comprising ShReD_42,50_. (**D**) Reaction-to-reaction distances calculated using the fluxes shown in panel (**A**) and equation 3. Note that ShReD_42,50_ for the Day 12 model involves only R_42_ and R_50_, as the contribution of R_50_ to the CoA flux is significantly larger compared to the Day 4 mode.

In equation 3, the index k refers to the set of reactions R_k_ that produce the intermediary metabolite M, v_k_ is the flux of R_k_, and v_j_ is the flux of the reaction R_j_. When *v*_j_ is close to zero, the corresponding edge distance is very large, as is any ShReD that includes this edge. In such cases, allowing a ShReD to reach an arbitrarily large value could exaggerate the numerical difference between reactions whose fluxes are not statistically different from zero. Therefore, the value of a flux-weighted ShReD was capped with an upper bound. For numerical convenience, the cap was set at 100, as fewer than 5% of all ShReDs calculated in this study exceeded this value. The calculation of ShReDs based on flux weights is illustrated in Figure 8. Distinct flux distributions (Figures 8A and 8C) can result in different ShReDs for the same reaction pair (Figures 8B and 8D).

### Partition algorithm

Partitions of flux-weighted and unweighted network models were generated using Newman’s community detection algorithm [28] similar to our previous work. The overall algorithm flow is shown in Figure S3 (Additional file 1). Briefly, the partitioning algorithm begins by finding the connected subnetworks in the parent network using a breadth-first traversal algorithm [29], as it is possible that the parent network, represented as a reaction centric graph, may not be fully connected. Each connected subnetwork is then partitioned into two daughter subnetworks to maximize a modularity score. Applied recursively, the algorithm produces a hierarchical tree of modules. Unlike our previous work, we do not require each daughter subnetwork to contain at least one cycle as a criterion for partition. It is sufficient that at least one daughter subnetwork contains at least one cycle. This relaxation allows the algorithm to find solutions (reaction node assignments) that result in a partition where single reaction nodes peel off from a larger subnetwork. While the single reaction nodes obviously cannot possess a cycle, this should not preclude further partitioning of the larger subnetwork.

#### Modularity matrix

In our previous work, we computed a modularity score based on the difference between the actual and expected ShReD assuming that each edge in the graph model has equal length, or weight. In this study, we modify the modularity matrix from which the modularity score is calculated to account for the skewness of weighted ShReD distributions. The desired modularity matrix **V** has a positive entry V_ij_ if the corresponding ShReD between a pair of reaction nodes is small relative to the expected ShReD, whereas it has a negative entry if the corresponding ShReD is large. Due to the skewing effect of the flux weights on the ShReD distribution, the determination of whether a weighted ShReD is small or large relative to expectation was based on a log ratio. Formally, we define an entry V_ij_ in the modularity matrix **V** as follows.

(4) $$V_{\mathrm{ij}}=\ln\left(\frac{p_{\mathrm{ij}}}{1-p_{\mathrm{ij}}}\right)$$

In equation 4, p_ij_ is the fraction of all weighted ShReDs involving reaction R_i_ or R_j_ that is longer than the ShReD between R_i_ and R_j_ (ShReD_ij_). If exactly half of all ShReDs involving R_i_ or R_j_ are longer (or shorter) than ShReD_ij_, then V_ij_ is zero. Otherwise, V_ij_ is positive or negative depending on the rank of ShReD_ij_ relative to all other ShReDs involving R_i_ or R_j_. As an example, consider the subnetwork shown in Figure 2B (module #7249). The flux weighted ShReD matrix for this subnetwork is shown in Figure S4 (Additional file 1). There are a total of 26 ShReDs involving R24 or R31, including the ShReD between R24 and R31 (ShReD_24,31_). Of these, ShReD_24,31_ ranks 11th in terms of length. Applying equation 4, p_24,31_ = 10/25 = 0.4, and V_24,31_ = −0.41. If p_ij_ = 0, p_ij_ is arbitrarily set to 0.01. The smallest V_ij_ value is thus −4.60, which is on the same order of magnitude as the other entries in the modularity matrix.

#### Optimization of modularity score

To generate a partition, we assign each reaction in a subnetwork into one of two daughter subnetworks. The goal is to find a set of assignments, represented by a binary vector **s**, that maximizes the modularity score [28]. The sum score is defined based on the modularity matrix **V**:

(5) $$Q=\sum_{i}\sum_{j}V_{\mathrm{ij}}s_{i}s_{j}=\mathrm{sV}s^{T}$$

Each element s_i_ or s_j_ of vector **s** has a value of either −1 or 1. An increase in Q is obtained in two cases: if V_ij_ is positive and reactions i and j are assigned to the same subnetwork (s_i_ = s_j_ = 1 or s_i_ = s_j_ = −1), or if V_ij_ is negative and the two reactions are assigned to different subnetworks (s_i_ = 1 and s_j_ = −1 or vice versa). A solution to the maximization problem can be found using a number of different optimization methods. For example, an approximate solution can be obtained using eigenvalue decomposition [28]. In this study, we used a genetic algorithm (GA). While the GA was computationally less efficient than the eigenvalue decomposition method, it yielded superior solutions (**s** vectors) with larger Q scores. The GA was implemented using custom code written in MATLAB with the following parameters. The initial population of solutions comprised 100 randomly generated **s** vectors. The population size was kept constant. A fixed fraction (60%) of the solutions was selected for reproduction based on fitness (Q score). New individuals were bred through crossover and mutation. During crossover, an element in the offspring **s** vector was assigned the same value as the corresponding elements in the parent **s** vectors if the values were the same in both parents. Otherwise, the element was randomly assigned either −1 or 1. The mutation (sign change) rate was set to 20%. The GA terminated when the average Q score of the population reached a plateau with an absolute slope < 0.05 with respect to generation number. The fittest solution (**s** vector with the largest Q score) generated over the course of the GA was used for the partition. For the subnetworks encountered in this study, termination was reached generally within 200 generations. For the example subnetwork of Figure 2B (Module #7249), the GA terminated in 117 generations, and clearly outperformed the eigenvalue solution (Additional file 1: Figure S5). In cases where the subnetwork size was sufficiently small (< 9 reactions), an exhaustive search was performed to find a globally optimal solution. The runtime for the complete partitioning of the Day 12 model was 180 seconds using the GA and 85 seconds using the eigenvalue approximation on a laptop computer with a 2.2 GHz CPU (Intel Core 2 Duo) and 4 GB of physical memory.

### Hierarchical tree of modules

The partitioning results are reported in the form of a hierarchical tree annotated with several properties. Each module is represented as a pie chart, where the size of each slice is proportional to the fraction of reactions that belong to the corresponding, pre-assigned canonical (textbook) grouping. The homogeneity index of a module corresponds to the fraction occupied by the largest slice in the pie chart. The homogeneity index therefore ranges from 0 to 1, where a larger number indicates greater homogeneity in terms of composition based on the canonical group assignments. The black lines connecting the nodes in the hierarchical tree represent ShReD-based partitions, whereas the red lines represent the formation of components from partitions that include disconnected components. The depth of a module is determined as the number of black edges traversed from the root node to the module. The height of a module is determined as the largest possible number of black edges traversed from the module to a terminal leaf node.

### Partition score for reaction pairs

To determine the correlation between modularity and flux weighted ShReD-based partitioning, we define a partition score H for a pair of reaction nodes by scaling the number of shared modules in the partition tree with respect to the partition depth of the terminal modules for the reaction nodes:

(6) $$H_{\mathrm{ij}}=\frac{\mathrm{Shared}-1}{2}\left[\frac{1}{m_{i}}+\frac{1}{m_{j}}\right]$$

where *Shared* is the number of modules in the partition hierarchy that include both reactions i and j, and m_i_ and m_j_ are, respectively, the maximal depth of reactions i and j. The numerical range of H is thus from 0 to 1. A value of zero indicates that the two reactions are immediately separated after the first partition operation, whereas a value close to one indicates that the two reactions remain together in the same module through many rounds of partition operations.

### Reaction pair H-V space Euclidean distance

To assess the impact of metabolic state and its corresponding flux distribution on the hierarchical partition of reaction modules, a Euclidean distance is computed for each reaction pair in the H-V (partition score – modularity score) coordinate space from its original location corresponding to the first metabolic state to its new location corresponding to the second state. All coordinates are normalized to the mean partition score and modularity score of the corresponding flux-weighted partition.

## Competing interests

The authors do not report any financial or other commercial conflict of interest.

## Authors’ contributions

Conceived and designed the experiments: GVS SH KL. Performed the experiments: GVS MY SH. Analyzed the data: GVS MY SH KL. Contributed reagents/materials/analysis tools: GVS SH KL. Wrote the paper: GVS MY SH KL. Wrote the program code used in the analysis: GVS SH. All authors read and approved the final manuscript.

## Supplementary Material

Additional file 2 **Reaction definitions and composition of each module in Figures **2** and **5**.** Click here for file

Additional file 1 **Includes Tables S1-S2 and Figures S1-S5.** The tables and figures are referenced in the text. Click here for file
