# Supplementary material for: Metabolic Flux-Based Modularity using Shortest Retroactive distances
Source: BMC Syst Biol. 2012 Dec 27;6:155. doi: 10.1186/1752-0509-6-155 (PMC3556310; doi:10.1186/1752-0509-6-155)
Supplement: Additional file 1 — Includes Tables S1-S2 and Figures S1-S5. The tables and figures are referenced in the text. [file 1752-0509-6-155-S1.docx]

**Table S1: Adipocyte model reaction definitions**

| Reaction | Stoichiometry |
| --- | --- |
|  | Transport |
| 1 | Glucose-E 🡪 Glucose |
| 2 | Lactate 🡪 Lactate-E |
| 3 | Glycerol 🡪 Glycerol-E |
| 4 | Palmitate 🡪 Palmitate-E |
| 5 | TG 🡪 TG-E |
| 6 | Alanine-E 🡪 Alanine |
| 7 | Aspartate-E 🡪 Aspartate |
| 8 | Asparagine-E 🡪 Asparagine |
| 9 | Glutamine-E 🡪 Glutamine |
| 10 | Glutamate-E 🡪 Glutamate |
| 11 | Glycine-E 🡪 Glycine |
| 12 | Proline-E 🡪 Proline |
| 13 | Serine-E 🡪 Serine |
| 14 | Tyrosine-E 🡪 Tyrosine |
| 15 | Histidine-E 🡪 Histidine |
| 16 | Isoleucine-E 🡪 Isoleucine |
| 17 | Leucine-E 🡪 Leucine |
| 18 | Lysine-E 🡪 Lysine |
| 19 | Methionine-E 🡪 Methionine |
| 20 | Phenylalanine-E 🡪 Phenylalanine |
| 21 | Threonine-E 🡪 Threonine |
| 22 | Valine-E 🡪 Valine |
| 23 | O_2_-E 🡪 O_2_ |
|  | Carbohydrate Metabolism |
| 24 | Glucose + ATP 🡪 Glucose 6-Phosphate + ADP |
| 25 | Glucose 6-Phosphate 🡪 Fructose 6-Phosphate |
| 26 | Fructose 6-Phosphate + ATP 🡪 Glyceraldehyde 3-Phosphate + ADP |
| 27 | Glycerone-Phosphate 🡪 Glyceraldehyde 3-Phosphate |
| 28 | Glyceraldehyde 3-Phosphate + NAD + ADP 🡪 3-Phosphoglycerate + NADH + ATP |
| 29 | 3-Phosphoglycerate 🡪 Phosphoenolpyruvate |
| 30 | Phosphoenolpyruvate + ATP 🡪 Pyruvate + ADP |
| 31 | Pyruvate + NADH 🡪 Lactate + NAD |
| 32 | Glucose 6-Phosphate + 2 NADP 🡪 2 NADPH + Ribulose 5-Phosphate |
| 33 | Ribulose 5-Phosphate + Erythrose 4-Phosphate 🡪 Fructose 6-Phosphate + Glyceraldehyde 3-Phosphate |
|  | TCA Cycle/ Oxidative Phosphorylation |
| 34 | Pyruvate-M + Oxaloacetate-M + NAD-M 🡪 NADH-M + Citrate-M |
| 35 | Pyruvate-M + ATP 🡪 Oxaloacetate-M + ADP |
| 36 | Citrate-M + NAD-M 🡪 2-Oxoglutarate-M + NADH-M |
| 37 | 2-Oxoglutarate-M + CoA + NAD-M 🡪 NADH-M + Succinyl-CoA-M |
| 38 | Succinyl-CoA-M + ADP 🡪 CoA + Succinate-M + ATP |
| 39 | FAD-M + Succinate-M 🡪 FADH_2_-M + Fumarate-M |
| 40 | Fumarate-M 🡪 Malate-M |
| 41 | Malate-M + NAD-M 🡪 NADH-M + Oxaloacetate-M |
| 48 | NADH-M + 0.5 O_2_ + 2.3 ADP 🡪 2.3 ATP + NAD-M |
| 49 | FADH_2_-M + 0.5 O_2_ + 1.4 ADP 🡪 1.4 ATP + FAD-M |
|  | Lipid Metabolism |
| 46 | Oxaloacetate + ATP 🡪 Phosphoenolpyruvate + ADP |
| 47 | Phosphoenolpyruvate + 2 ATP + 2 NADH 🡪 Glycerone-Phosphate + 2 NAD + 2 ADP |
| 50 | 8 Acetyl-CoA + 14 NADPH + 7 ATP 🡪 8 CoA + 14 NADP + Palmitate + 7 ADP |
| 51 | Glycerone-Phosphate + 3 Palmitate + NADH 🡪 NAD + Tripalmitoylglycerol |
| 52 | Tripalmitoylglycerol 🡪 3 Palmitate + Glycerol |
|  | Citrate/ Malate Shuttle |
| 42 | Citrate + CoA + ATP 🡪 Acetyl-CoA + Oxaloacetate + ADP |
| 43 | Oxaloacetate + NADH 🡪 Malate + NAD |
| 44 | Malate + NADP 🡪 NADPH + Pyruvate |
| 45 | Citrate + NADP 🡪 2-Oxoglutarate + NADPH |
| 53 | Pyruvate 🡪 Pyruvate-M |
| 54 | Citrate-M 🡪 Citrate |
| 55 | 2-Oxoglutarate + Malate-M 🡪 2-Oxoglutarate-M + Malate |
|  | Amino Acid Metabolism |
| 56 | Pyruvate + Glutamate 🡪 2-Oxoglutarate + Alanine |
| 57 | ATP + Aspartate + Glutamine 🡪 Glutamate + Asparagine |
| 58 | Oxaloacetate + Glutamate 🡪 2-Oxoglutarate + Aspartate |
| 59 | Glutamine 🡪 Glutamate |
| 60 | 2-Oxoglutarate + NADH 🡪 NAD + Glutamate |
| 61 | Serine 🡪 Glycine |
| 62 | Serine 🡪 Pyruvate |
| 63 | 2 NADH + Glutamate 🡪 2 NAD + Proline |
| 64 | Phenylalanine 🡪 Tyrosine |
| 65 | Tyrosine 🡪 Fumarate-M |
| 66 | Histidine 🡪 Glutamate |
| 67 | Isoleucine + 2 CoA 🡪 FADH_2_-M + Acetyl-CoA + 2 NADH-M + Succinyl-CoA-M |
| 68 | Leucine + ATP 🡪 Acetyl-CoA + FADH_2_-M + 2 NADH-M |
| 69 | Lysine + CoA 🡪 FADH_2_-M + 3 NADH + 2 NADH-M |
| 70 | Serine + Methionine + ATP + CoA 🡪 Acetyl-CoA + NADH |
| 71 | Threonine + CoA 🡪 Acetyl-CoA + NADH + Glycine |
| 72 | Valine + CoA 🡪 FADH_2_-M + 4 NADH + Succinyl-CoA-M |

Suffixes E and M refer to extracellular and mitochondrial metabolites, respectively.

**Table S2: Flux distributions**

| Reaction | Day 4 | Day 12 | Day 12 w/ LDH inhibition | Day 12 w/ PCX inhibition |
| --- | --- | --- | --- | --- |
| 1 | 268.0 | 266.0 | 212.0 | 341.0 |
| 2 | 359.7 | 200.0 | 119.0 | 428.0 |
| 3 | 5.0 | 8.6 | 19.8 | 20.6 |
| 4 | 0.3 | 1.1 | 1.0 | 0.8 |
| 5 | 0.9 | 4.8 | 2.3 | 2.9 |
| 6 | -15.4 | 0.7 | 0.6 | -0.5 |
| 7 | 0.0 | 0.0 | 0.0 | 0.0 |
| 8 | -2.4 | -3.0 | -1.1 | -4.3 |
| 9 | 4.3 | -11.2 | -12.0 | 4.3 |
| 10 | 1.5 | 0.4 | 0.4 | 0.3 |
| 11 | -8.8 | -7.2 | -5.3 | -6.8 |
| 12 | -1.2 | -0.4 | -0.5 | -2.1 |
| 13 | 9.2 | 5.7 | 4.6 | 5.7 |
| 14 | 0.6 | -0.4 | 0.0 | 0.0 |
| 15 | 2.5 | 0.0 | 0.0 | 0.0 |
| 16 | 9.5 | 8.2 | 6.1 | 7.5 |
| 17 | 10.6 | 8.8 | 6.8 | 7.9 |
| 18 | 2.8 | 0.3 | 0.0 | 0.0 |
| 19 | 0.8 | 0.0 | 0.0 | 0.0 |
| 20 | 0.6 | 0.4 | 0.0 | 0.0 |
| 21 | 0.8 | 1.6 | 0.6 | 1.1 |
| 22 | 5.8 | 5.5 | 3.3 | 3.8 |
| 23 | 499.0 | 726.2 | 662.7 | 617.0 |
| 24 | 268.0 | 266.0 | 212.0 | 341.0 |
| 25 | 265.8 | 209.1 | 186.6 | 300.2 |
| 26 | 268.0 | 266.0 | 212.0 | 341.0 |
| 27 | 472.3 | 628.0 | 483.4 | 580.9 |
| 28 | 742.5 | 950.9 | 720.9 | 962.7 |
| 29 | 742.5 | 950.9 | 720.9 | 962.7 |
| 30 | 532.3 | 655.6 | 462.6 | 736.6 |
| 31 | 359.7 | 200.0 | 119.0 | 428.0 |
| 32 | 2.2 | 56.9 | 25.4 | 40.8 |
| 33 | 2.2 | 56.9 | 25.4 | 40.8 |
| 34 | 178.8 | 375.7 | 305.1 | 280.3 |
| 35 | 1.1 | 137.8 | 69.6 | 63.7 |
| 36 | 161.2 | 224.2 | 226.2 | 205.4 |
| 37 | 183.6 | 259.0 | 243.3 | 223.3 |
| 38 | 198.9 | 272.7 | 252.7 | 234.6 |
| 39 | 198.9 | 272.7 | 252.7 | 234.6 |
| 40 | 200.1 | 272.7 | 252.7 | 234.6 |
| 41 | 177.7 | 237.9 | 235.6 | 216.7 |
| 42 | 2.4 | 105.4 | 49.7 | 59.5 |
| 43 | 0.0 | 22.3 | 13.3 | 18.0 |
| 44 | 22.4 | 57.2 | 30.5 | 35.9 |
| 45 | 15.3 | 46.1 | 29.3 | 15.5 |
| 46 | 0.0 | 80.1 | 35.3 | 37.3 |
| 47 | 210.2 | 375.4 | 293.5 | 262.4 |
| 48 | 762.6 | 1148.3 | 1049.6 | 972.3 |
| 49 | 235.3 | 304.0 | 275.7 | 261.7 |
| 50 | 3.0 | 15.5 | 7.9 | 9.5 |
| 51 | 5.9 | 13.4 | 22.1 | 23.5 |
| 52 | 5.0 | 8.5 | 19.8 | 20.6 |
| 53 | 180.0 | 513.5 | 374.7 | 344.0 |
| 54 | 17.6 | 151.5 | 79.0 | 75.0 |
| 55 | 22.4 | 34.9 | 17.2 | 17.9 |
| 56 | 15.4 | -0.7 | -0.6 | 0.5 |
| 57 | 2.4 | 3.0 | 1.1 | 4.3 |
| 58 | 2.4 | 3.0 | 1.1 | 4.3 |
| 59 | 1.9 | 14.2 | 13.1 | 0.0 |
| 60 | 10.7 | 13.5 | 12.6 | 2.3 |
| 61 | 8.0 | 5.7 | 4.6 | 5.7 |
| 62 | 0.4 | 0.0 | 0.0 | 0.0 |
| 63 | 1.2 | 0.4 | 0.5 | 2.1 |
| 64 | 0.6 | 0.4 | 0.0 | 0.0 |
| 65 | 1.2 | 0.0 | 0.0 | 0.0 |
| 66 | 2.5 | 0.0 | 0.0 | 0.0 |
| 67 | 9.5 | 8.2 | 6.1 | 7.5 |
| 68 | 10.6 | 8.8 | 6.8 | 7.9 |
| 69 | 10.6 | 8.8 | 6.8 | 7.9 |
| 70 | 0.8 | 0.0 | 0.0 | 0.0 |
| 71 | 0.8 | 1.6 | 0.6 | 1.1 |
| 72 | 5.8 | 5.5 | 3.3 | 3.8 |

All units are in mmol/g-DNA/2 days. Values shown are mean fluxes (n = 6). Fluxes for the Day 4 condition were calculated using data taken from (Si et al, 2007). Fluxes for all other conditions were calculated using data taken from (Si et al, 2009).

**
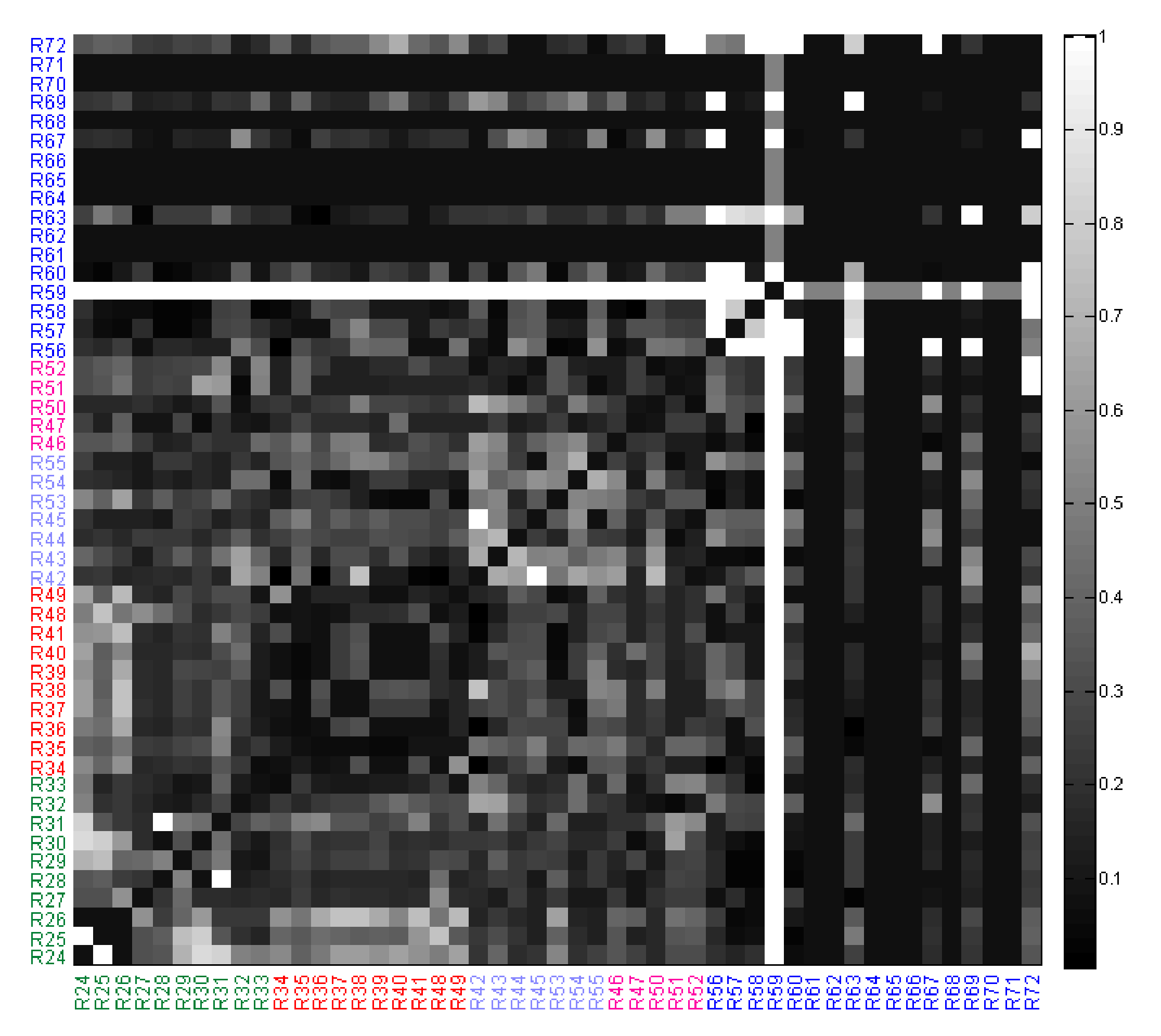
**

**Figure S1:** Heat map of the H-V Euclidean distance between the Day 12 model and Day 12 model with PCX inhibition. A dark square indicates that the corresponding reaction pair has similar modularity and partition scores in the two models, and thus is likely to be placed into similar modules in the two partition trees.

**
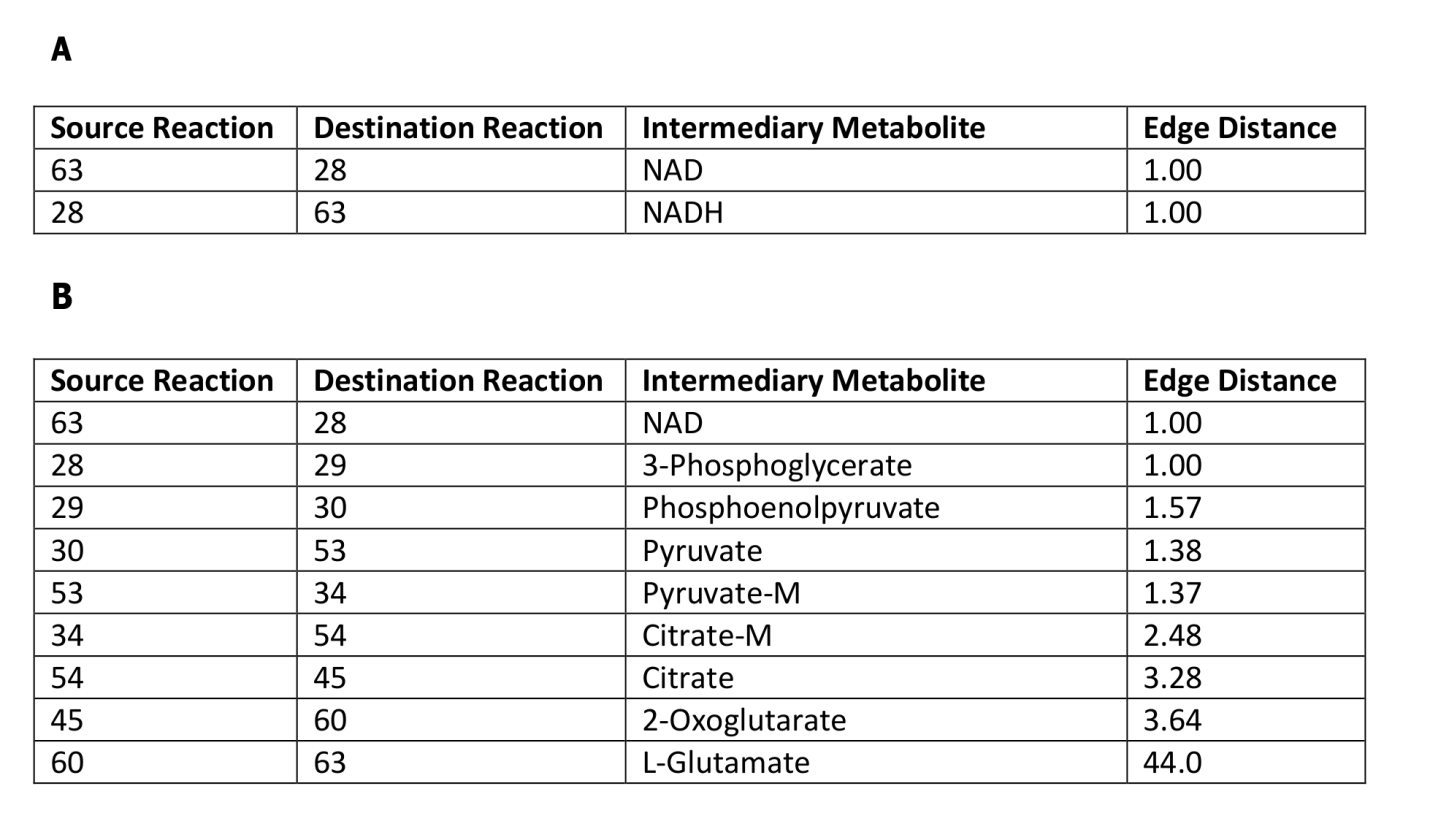
**

**Figure S2: (A)** ShReD of reaction pair [R28, R63] in the unweighted network is equal to 2.0. **(B)** ShReD of the same reaction pair for the Day 12 weighted network is equal to 60.

**
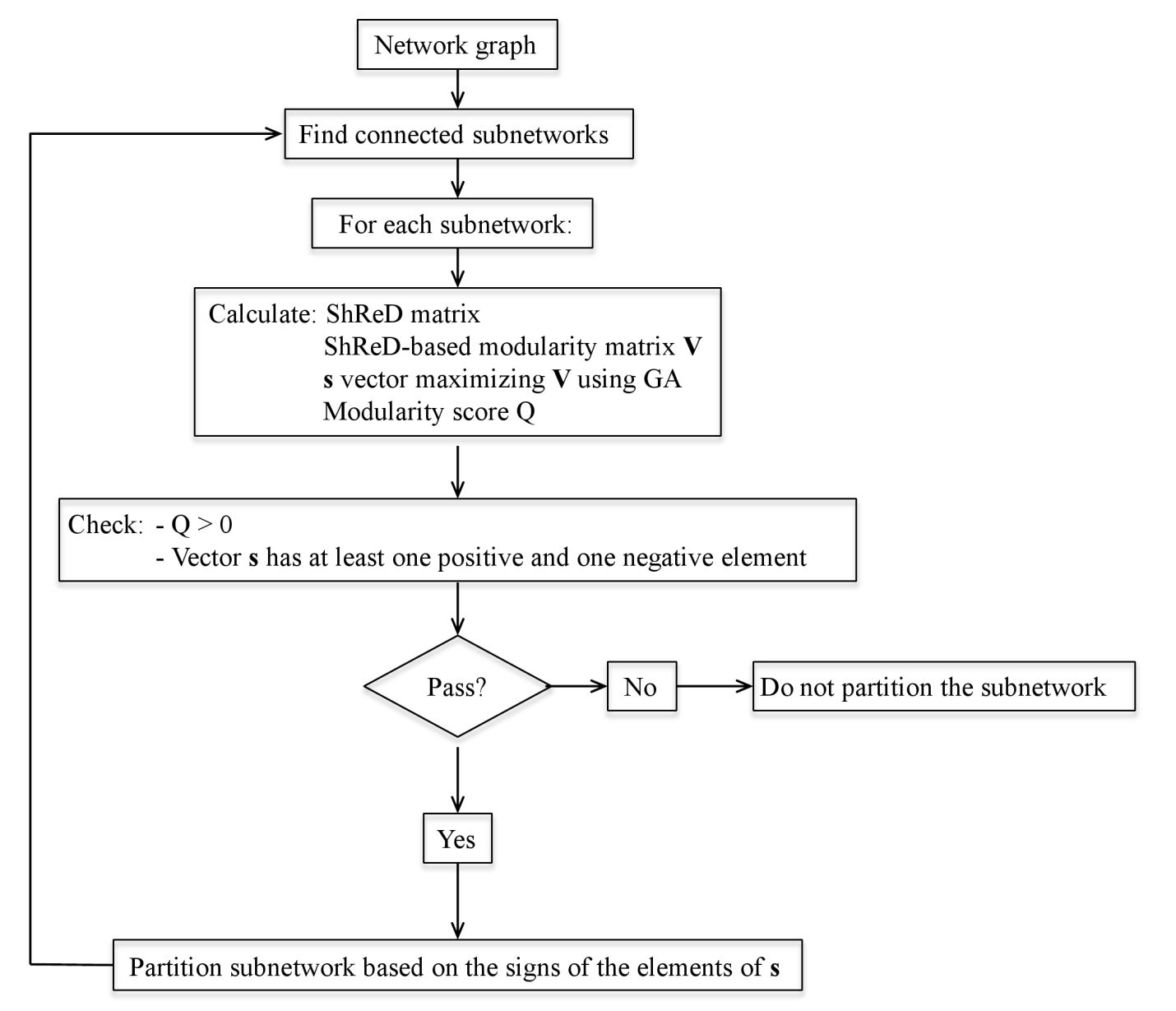
**

**Figure S3:** Algorithm workflow for ShReD-based partitioning using flux-weighted edges.

**
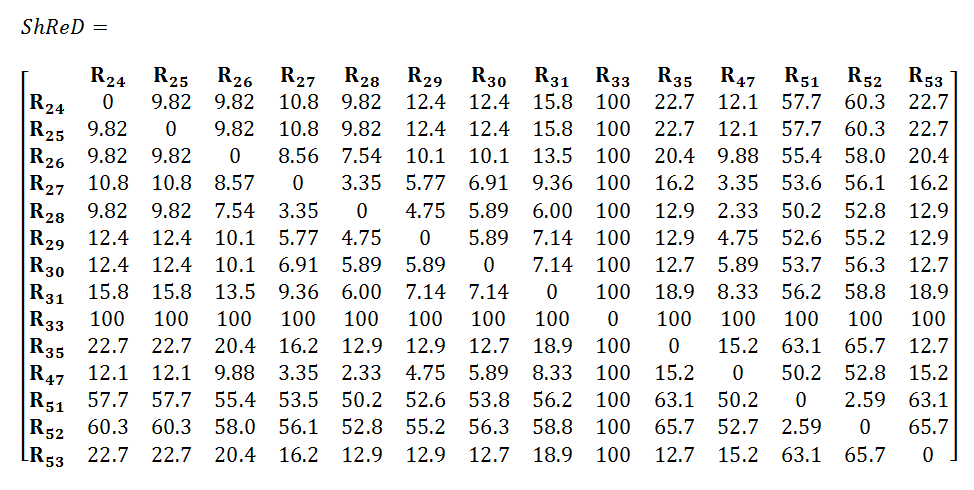
**

**Figure S4:** ShReD matrix of module #7249 in the Day 12 partition.

**
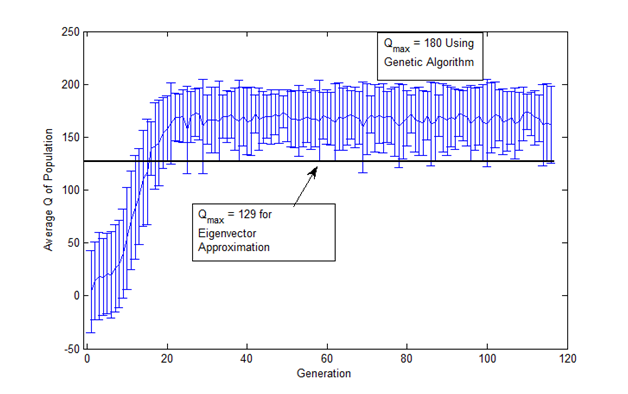
**

**Figure S5:** Genetic algorithm (GA)-based optimization of the modularity score (Q). Plot shows the evolution of the fitness function Q as a function of generation number. The maximal Q score obtained using the eigenvector approximation (Q_max_ = 129) is indicated with the bold horizontal line. In the example shown, which refers to module #7249 in the Day 12 partition, the optimal GA solution (Q_max_ = 180) is reached after ~20 generations.
